# Immersive Molecular Dynamics in Virtual Reality: Increasing Efficiency of Educational Process with Companion Converter for NarupaXR

**DOI:** 10.3390/jimaging7060097

**Published:** 2021-06-08

**Authors:** Polina Pereshivkina, Nadezhda Karandasheva, Maria Mikhaylenko, Mikhail Kurushkin

**Affiliations:** Chemistry Neuroeducation Laboratory, SCAMT Institute, ITMO University, 9 Lomonosova Str., 191002 Saint Petersburg, Russia; pereshivkina@scamt-itmo.ru (P.P.); karandasheva@scamt-itmo.ru (N.K.); mikhailenko@itmo.ru (M.M.)

**Keywords:** Narupa, NarupaXR, converter, xml file, mol2 file, virtual and augmented reality, chemistry education

## Abstract

Visualization has always been a crucial part of the educational process. Implementing computer algorithms and virtual reality tools into it is vital for the new generation engineers, scientists and researchers. In the field of chemistry education, various software that allow dynamic molecular building and viewing are currently available. These software are now used to enhance the learning process and ensure better understanding of the chemical processes from the visual perspective. The present short communication provides a summary of these applications based on the NarupaXR program, which is a great educational tool that combines the functionality and simple design necessary for an educational tool. NarupaXR is used with a companion application “Narupa Builder” which requires a different file format, therefore a converter that allows a simple transition between the two extensions has been developed. The converter sufficiently increases the efficiency of the educational process. The automatic converter is freely available on GitLab The current communication provides detailed written instructions that can simplify the installation process of the converter and facilitate the use of both the software and the hardware of the VR set.

## 1. Introduction

Due to the rapid development of computer technologies, more attention is paid to the areas of their intersection with chemistry, including infochemistry [1,2,3,4]. In order to prepare the 21st century students to be successful researchers and engineers in emerging fields of chemistry, informatics and programming, it is necessary to provide more educational tools to ensure deeper understanding of the fundamental principles in the aforementioned areas. Computer algorithms are available for working with geometric aspects of structural chemistry and materials science [4]. Unique platforms are accessible for the purpose of molecular visualization and design as well as molecular viewing software where molecules can be animated [5,6]. New interactive tools are being created in order to improve the quality of teaching and open up plenty of opportunities for users to “interact” with real molecules (gases), understand their behavior and computationally simulate dynamic processes [7,8,9,10]. Moreover, it has been recently reported in various studies that point atomic models in electron microscopy and X-ray density maps were built using virtual reality [11]. Other studies have reported that two major motivations for the use of computer technologies in teaching process are (1) visualization of the molecular world and (2) dynamic simulation of its behavior. Simulations that allow users to interact with molecules have recently become technically available and already have been successfully applied in chemistry education [12,13].

Currently, the most prominent programs for working with molecules in virtual reality include UCSF ChimeraX [11,14], Molecular Zoo, AltPDB [11] and NarupaXR [15,16]. All of these programs are based on the Unity 3D video game engine, which does not itself have a built-in support for molecules. However, there is a special library UnityMol, which allows to display molecules in Unity3D. Regarding the programming languages, Molecular Zoo and NarupaXR are written in C #, AltPDB is written in JavaScript, while ChimeraX is written in a combination of Python and C ++.

AltPDB is not a dynamic platform; its main advantage is the possibility of an online lesson with multiple people or a whole class present at the same time. Molecular Zoo is mainly used to present simple and familiar biomolecules in dynamics, where several molecules can be launched simultaneously, their collisions can be observed [11]. Although these two programs can be successfully implemented into the chemistry education curriculum, they lack complexity since students cannot build molecules and bonds from scratch.

From the user’s point of view, NarupaXR and ChimeraX share more similarities than any other two programs, since they are both focused on molecular dynamics. NarupaXR is a software for observations of molecular dynamics. Furthermore, Narupa Builder is a separate application that allows building new molecules and should be used in combination with NarupaXR. ChimeraX is a versatile molecular modeling toolkit for interactive visualization and analysis of molecular structures and related data, which was originally intended to visualize, interpret and manipulate only atomic-scale data [17]. Now it is also used for computational studies of molecules, macromolecular assemblies and their dynamics. It is worth noting that ChimeraX was originally developed for more complex tasks, such as analyzing molecular structures and electron and light microscopy data, while NarupaXR is aimed at teaching school and university students [11]. Therefore, the main advantage of NarupaXR is the absence of an overcrowded interface with many functions that are not relevant for the students. NarupaXR presents the perfect combination of minimalism in design and high-quality functionality. Hence, NarupaXR is the most suitable software for the particular purpose of this research.

The major challenge that NarupaXR users can face is the creation of their own simulations, which has to be conducted in a different program. Narupa Builder, a companion application to NarupaXR, which allows building, design and editing of molecules in VR with the help of HTC Vive controllers. Narupa Builder has a convenient “minimization” feature where, once the molecule is constructed, the distances between the atoms are adjusted automatically. However, at the next step, the user has to perform a substantial amount of manual programming to transform the mol2 files that mainly consist of the atoms’ coordinates into lengthy xml files that can be loaded into NarupaXR to run the simulations.

Thus, the goal of this work was to create an open access converter, which speeds up the process of transforming raw mol2 files, obtained from Narupa Builder, into the final xml files that are loaded into the NarupaXR environment. The current available converters require additional extensions in order to convert between these two formats, therefore creating a companion converter reduces the solutions of this problem to one step.

## 2. Hardware

The Narupa developers have shared some of their recommendations to make a personal VR lab for n-person (they have successfully tested it on a maximum of *n* = 7) class.

For an n-person VR lab it is necessary to have n PCs and n HTC Vive (or alternatively HTC Vive Pro) headsets with the controllers. The instructions on how to connect all parts of the HTC Vive with PC are available in the package or via the official website.

The list of the necessary hardware is presented below:

1. An 8-port network switch such as: NETGEAR GS208-100UKS 8 Port Gigabit Ethernet Desktop Switch, only needed for WiFi connection less than 300 mb/s and more than one-person VR lab.
2. One computational server, which will operate as the “force field engine” and equipped with
  - GPU, for example, an NVIDIA 1080Ti;
  - CPU, for example, an AMD Ryzen 7 1700X sAM4 3.8 GHz;
  - disk space for storing real-time trajectory data;
  - n + 1 LAN cables.

More detailed information regarding the setup is available via the following link (https://gitlab.com/intangiblerealities/narupaXR/blob/master/DocumentationSource/UserGuide/UserGuide.md (accessed on: 1 January 2020)).

## 3. Software Tools and Functionalities

To work with molecules in virtual reality, it is necessary to install Narupa Builder and NarupaXR front end. To use the programs, downloaded files must be unzipped into one folder. For HTC Vive to work, the nVidia driver needs to be updated to the latest version, it is recommended to download the HTC Vive driver from the official website. If the Steam and Steam VR software are not installed, download these programs to launch the HTC Vive. It is necessary to have a Steam login, which can be used on all computers in case of multiple users. In order to enable VR, Steam needs to be activated. Steam VR does not need to be launched separately, it will be launched automatically.

After installing motion sensors and starting Steam, the user will be automatically promoted to calibrate the equipment. The system describes the calibration process step by step in a simple and accessible way at the point of installation. To connect to the main server through which the latest version of NarupaXR will be launched, the developers recommend downloading launcher “itch” p).

We recommend downloading Visual Studio Code to create xml files, which are used to run simulations and install IntelliJ IDEA to run the special converter from the GitLab.

## 4. Materials and Methods

The starter version of NarupaXR offers only a few standard simulations (buckyballs, nanotube and methane, 17-alanine: knotted protein MJ0366 and H7N9 neuraminidase and Tamiflu). Some molecules have been built by developers and uploaded to the server, other molecules were built by us and are available via GitLab. In order to work with other structures that are not available on any of the aforementioned platforms, the user has to create them from scratch with the help of NarupaBuilder.

NarupaBuilder is a companion application to NarupaXR where molecules can be built (by adding new atoms, bonds), moved in space and the distance between them can be adjusted. It can be conducted with the use of special HTC controllers.

After unzipping the required file, Narupa Builder program can be launched by clicking on the icon. Since this is a local constructor, it does not require additional connection to servers, which allows building molecules without Internet connection. After launching the program, VR headsets and VR controllers can be put on. Initially, a grey screen will be visible; to build the molecules the controller has to be used.

Using the controllers, students can add and remove new atoms and bonds, as well as move molecules and their components in relation to each other. These actions are not difficult to perform, since each controller has its own menu, where the desired atom can be selected as well as the actions, which can be applied to it.

**Figure 1:**
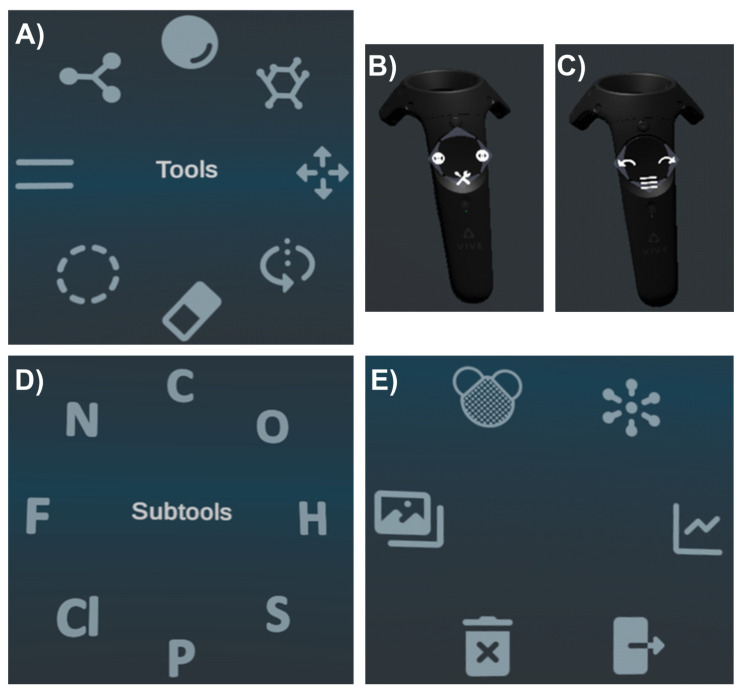
(**A**) the menu of the right controller; (**B**) the buttons of the right controller; (**C**) the menu of the subtools on the right controller; (**D**) the menu of the subtools on the right controller; (**E**) the menu of the subtools on the right controller.

First, the right controller will be described. To see the menu with the available actions, one needs to click on the bottom part of the circular panel (there is a wrench), the menu with tools is presented in Figure 1A,B.

By holding down this button, one can hover over various functions to see their name and functionality. The available tools are: Delete—deletes a single element that is chosen by hovering over it; Rotate—rotates some molecules relative to others; Move—moves atoms; Build Fragment—a previously saved fragment can be added by moving the desired fragment to the Users Fragments folder and choosing this option; Build Atom—opens a panel for selecting the atom to build; Connect—builds chemical bonds between individual atoms or fragments of the molecule; Bond Order—creates double bonds, triple bonds, etc.

Moreover, there is a very useful function, which shows where the chemical bond can be created in green and the bonds that are not available or chemically correct in red. One more function is to choose an atom from the Subtools menu, which is available when holding down the top button on the circle panel. The Subtools menu is presented in Figure 1D.

Now the functions of the second controller will be described. More specifically, when the bottom part of the circular panel of the left controller is clicked (there are three horizontal stripes), a menu appears: Figure 1C,E.

By holding down this button and hovering over various functions, one can see their names and understand what they are used for accordingly. The available functions are: Export—saves data; Minimize—reduces or increases bond lengths between atoms to the required size; Balance Hydrogens—aligns hydrogen bonds; Show/Hide Hydrogens—hides/shows hydrogens for a better view of other atoms and bonds; Toggle Image—opens up a folder with necessary pictures, which show the detailed structure of the desired molecule and can be used as a hint when building molecules; Delete All—removes all atoms and bonds that have been built.

After the molecule is completed, the file should be exported with the help of the left controller. As a result, the mol2 file with code of molecule will be obtained, but in order to run it in the NarupaXR, first it should be converted to xml format.

For more detailed video tutorials with the description of NarupaXR functions one can visit the developer’s website (https://vimeo.com/intangiblerealities (accessed on 1 January 2020)).

Let us consider ethane as an example. The initial code that can be retrieved from Narupa Builder (after the export) is:

@<TRIPOS>MOLECULE

narupa builder molecule

8 7 0 0 0

SMALL

GASTEIGER

@<TRIPOS>ATOM

1 C1 -6,165 0,925 -6,713 C.3 1 N1

2 C2 -5,555 0,741 -5,798 C.3 1 N1

3 H1 -7,070 0,516 -6,599 H 1 N1

4 H2 -5,711 0,516 -7,504 H 1 N1

5 H3 -6,261 1,910 -6,857 H 1 N1

6 H4 -4,737 0,221 -6,042 H 1 N1

7 H5 -6,096 0,221 -5,137 H 1 N1

8 H6 -5,287 1,615 -5,394 H 1 N1

@<TRIPOS>BOND

1 2 1 1

2 8 2 1

3 7 2 1

4 6 2 1

5 5 1 1

6 4 1 1

7 3 11

## 5. Results and Discussion

In the xml file, which can be run in NarupaXR, it is necessary to type all coordinates, temperature, types of bonds and other structural information into a new xml file. For simple molecules it is fast to do by hand but with larger structures it takes a lot of time. Although, molecules as large as glucose or caffeine can be assembled and optimized manually, larger molecules are extremely time-consuming to assemble in this way and yet it is not guaranteed that the simulation will eventually work, due to a high possibility of a misspelling. These difficulties prompted the creation of new automatization strategies for the most optimal and user-friendly experience with Narupa Builder and NarupaXR. The results of this research is an open access converter, which sufficiently speeds up the process of transforming the raw mol2 files, obtained from Narupa Builder, into the final xml files that are loaded into the NarupaXR environment. The code was created for the purpose of research, engineering and especially education. The converter is publicly available via the following link (https://gitlab.com/teamSCAMT/converter_mol2_to_xml.git (accessed on 1 August 2020)). 

The converter reported in the present paper is a standalone code (however, it is not an executable); hence, in order to use it, several simple steps need to be taken. Further, these steps will be described in detail. For an illustrative guide it is recommended to address a brief illustrative description in the Supplementary Materials (The following are available online at www.mdpi.com/article/10.3390/jimaging7060097/s1).

First of all, one needs to create an account on a GitLab platform. Next, find the account “teamSCAMT” and the project called “converter_mol2_to_xml”. Then click on the “Fork” button.

To run the code, install the development environment. The recommended environment is the “IntelliJ IDEA”.

After installation, open the program and create a new project from version control. After that, paste the copied link in the URL line. In order to run the code with the help of IntelliJ IDEA application, one needs to open all the three classes of the code: “Reader”, “Beliviks” and “Writer”, this can be performed by clicking on them.

As a result, the folder with the converter code will appear on the desktop. Next, add all Narupa Builder mol2 files, which require conversion to the folder with the converter code. If everything is conducted right, the necessary mol2 file will appear in the IntelliJ IDEA between “Writer” and “README.md” classes.

**Figure 2:**
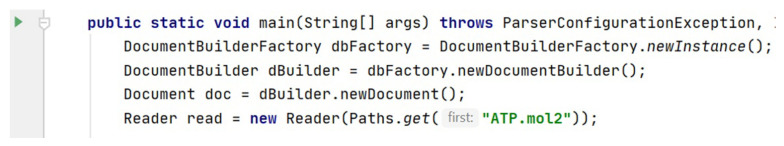
Opening the “Writer” class and indicating the name of the desired mol2 file.

Next, open class “Writer” and specify the name of the file, which requires conversion. An example is in Figure 2 in bold green (“ATP.mol2”).

**Figure 3:**
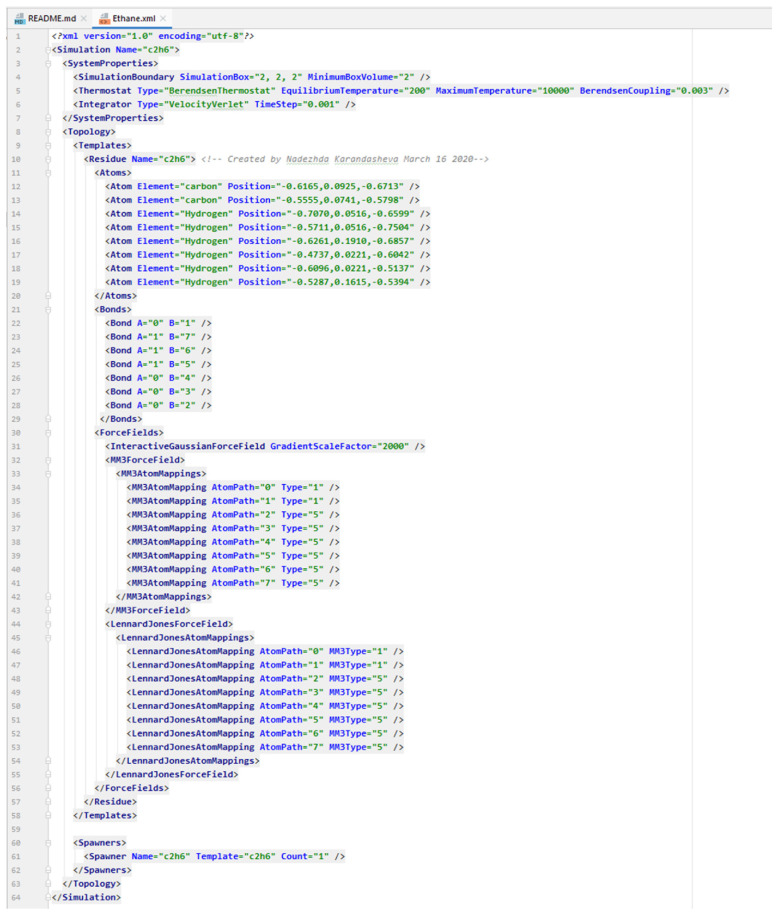
The example of the code converted into xml format.

Finally, run the code and the resulting xml code will appear in the output file. The converted into final xml file code is presented below in Figure 3.

In the final xml file code, there are parts, which the user needs to fill in manually. These are responsible for the assignment of bond type and are given in bold as can be seen in an example below. In order to assign the bond type, one needs a glossary provided in the mm3.xml file in the \server\Assets\Data\folder.

<MM3AtomMapping AtomPath = “0” **Type = “ ”**/>

<MM3AtomMapping AtomPath = “1” **Type = “ ”**/>

…

<MM3AtomMapping AtomPath = “7” **Type = “ ”**/>

…

<LennardJonesAtomMapping AtomPath = “0” **MM3Type = “ ”**/>

<LennardJonesAtomMapping AtomPath = “1” **MM3Type = “ ”**/>

…

<LennardJonesAtomMapping AtomPath = “7” **MM3Type = “ ”**/>

Before starting simulations, it is necessary to transfer the converted data file after Narupa Builder to the «Simulations» folder, which is located in the main folder containing all the unzipped files. To find it, open the folder: Server/Assets/Simulations.

There are two ways to launch NarupaXR program: local and via the international server. To launch locally, simply open the application and follow the launch instructions above. When choosing this method, there is a possibility to connect several people in one simulation, and this is possible even without using the Internet. This type of launch will be faster, but will not support the latest updates of this program, which, on the other hand, will be visible and available for use when launched through an international server.

**Figure 4:**
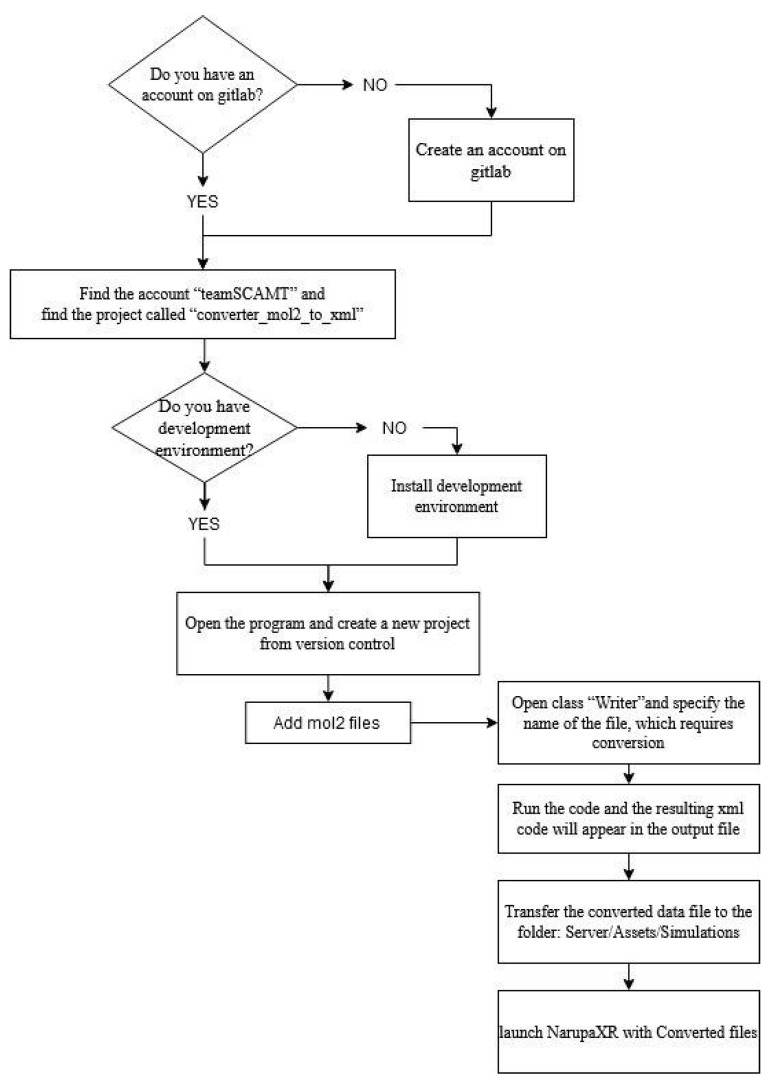
A step by step schematic diagram describing the usage of converter.

To run through a remote server, open the previously downloaded launcher “itch”, sign in or register there and find NarupaXR in the search bar in the store section. Make sure that NarupaXR is added to one’s game library. Click on the NarupaXR in the game library and then on the button “Launch”. After this the program will provide several ways to start the server, so click on the top option such as “Launch Server and VR” and quickly choose a desired simulation (from your computer, which you want to start) among folders on a personal computer. It is recommended to choose the simulation quickly (in less than a minute), otherwise the NarupaXR will start without any simulations and will have to be reloaded NarupaXR. A schematic diagram of the described installation process is presented on Figure 4.

Once the application is launched, and the data is received from the Narupa Builder app, it needs to be converted. Then it is necessary to launch the created simulations directly in NarupaXR. To perform this, the server called “narupa-server” (application) needs to be started, which is located in the folder “Server”. Then in the main unzipped folder, open the “NarupaXR” folder and start the “narupaXR” application. After that, a menu will appear on the screen (and in the headsets) in which one needs to first select “Browse Servers” and then “narupa-server” with the laser pointer on the right controller. 

**Figure 5:**
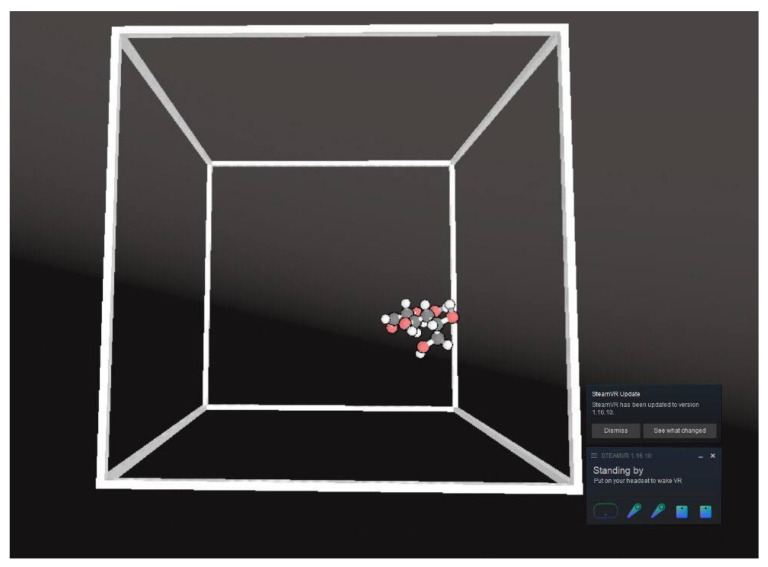
An example of the VR environment in NarupaXR showing d-glucose molecule.

Following that the headset can be put on, and the virtual box will appear with a yellow button on the left controller, which is visible when the eyes are moved towards the left hand. This button can be pressed to open the menu, then select the bottom button with the gears drawn on it and press “load simulations”, after that select the desired simulation. On the top of this menu the view option of the molecules can be chosen. The view option presented in the current paper is “Ball and Stick”, however others such as “Skeleton”, “VDW” are also available in the simulation. With the options in this menu, the user can pause, play and restart the simulations. An example of the simulation is presented on Figure 5.

## 6. Conclusions

In the present short communication we have developed a converter between mol2 and xml formats of the Narupa Builder and NarupaXR applications accordingly. The converter is freely available via GitLab. It can significantly improve the efficiency of the educational process using these two programs and avoid systematic human errors. The converter automates and facilitates the process of creating customized molecules, which otherwise would take hours. It allows customization of the laboratory course, with using not just molecular structures provided by the software, but also creating new ones. It could be used by the teaching assistant in preparation for the VR labs or by the students themselves. Moreover, we have provided a detailed description for the use of NarupaXR software, the converter and the VR headset, which makes implementation of these techniques as straightforward as possible. Virtual and augmented reality will inevitably change the way chemistry education is approached at the moment and enhance the process with visualizing concepts that are otherwise difficult to grasp. The converter presented in the current communication is a ready-to-use tool, which can accelerate these changes and make the field more modern, whilst ensuring effective teaching process.

## Data Availability

Not applicable.

## Supplementary Materials

The following are available online at https://www.mdpi.com/article/10.3390/jimaging7060097/s1. A brief illustrative description of how to use the converter. List of links for the discussed hardware and software (PDF).
