# Supplementary material for: Immersive Molecular Dynamics in Virtual Reality: Increasing Efficiency of Educational Process with Companion Converter for NarupaXR
Source: J Imaging. 2021 Jun 8;7(6):97. doi: 10.3390/jimaging7060097 (PMC8321342; doi:10.3390/jimaging7060097)
Supplement: Supplementary file 1 [file jimaging-07-00097-s001.zip › jimaging-1199357-supplementary.pdf]

Communication

Immersive Molecular Dynamics in Virtual Reality: Increasing Efficiency of Educational Process with Companion Converter for NarupaXR

Polina Pereshivkina, Nadezhda Karandasheva, Maria Mikhaylenko and Mikhail Kurushkin *

Chemistry Neuroeducation Laboratory, SCAMT Institute, ITMO University, 9 Lomonosova Str., 191002 Saint Petersburg, Russia; pereshivkina@scamt-itmo.ru (P.P.); karandasheva@scamt-itmo.ru (N.K.); mikhailenko@itmo.ru (M.M.)

* Correspondence: kurushkin@scamt-itmo.ru

Installing software to run NarupaXR:

Narupa Builder should be used in combination with NarupaXR, both of which should be installed from (https://irl.itch.io/narupaxr) and (https://irl.itch.io/narupa-builder). In order to use Narupa in VR lab, HTC Vive (or alternatively HTC Vive Pro) headsets with the controllers are necessary. The instructions on how to connect all parts of the HTC Vive with PC are available in the package or via the following link (https://www.vive.com/us/). nVidia driver needs to be updated to the latest version from the official website (https://www.nvidia.com/en-in/drivers/nvidia-update/). It is recommended to download the HTC VIVE driver from the official website (https://www.vive.com/us/setup/vive/). Download Steam and Steam VR software from the link (https://steamcommunity.com/steamvr) to launch the HTC VIVE.

Download launcher "itch" from the following link (https://itch.io/app). We recommend downloading Visual Studio Code (https://code.visualstudio.com/download) to create xml files. We recommend installing IntelliJ IDEA (https://www.jetbrains.com/idea/download/#section=windows) to run the special converter from the GitLab. Then, convert the mol2 files from Narupa Builder into xml files for the purpose of simulation.

In the xml file, which can be run in NarupaXR, it is necessary to type all coordinates, temperature, types of bonds and other structural information into a new xml file. For simple molecules, it is fast to do by hand, but with larger structures, it takes a lot of time. Although molecules as large as glucose or caffeine can be assembled and optimized manually, larger molecules are extremely time-consuming to assemble in this way and yet it is not guaranteed that the simulation will eventually work, due to a high possibility of a misspelling. These difficulties prompted the creation of new automatization strategies for the most optimal and user-friendly experience with Narupa Builder and NarupaXR. The results of this research are an open access converter, which sufficiently speeds up the process of transforming the raw mol2 files, obtained from Narupa Builder, into the final xml files that are loaded into the NarupaXR environment. The code was created for the purpose of research, engineering, in particular, education. The converter is publicly available via the following link (https://gitlab.com/teamSCAMT/converter_mol2_to_xml.git).

The converter reported in the present paper is a standalone code (however, it is not an executable); hence, in order to use it, several simple steps need to be taken. Thus, these steps will be described in detail.

First of all, one needs to create an account on a GitLab platform (https://gitlab.com/). Next, find the account "teamSCAMT" and the project called "converter_mol2_to_xml". Then, click on the "Fork" button (Figure S1).

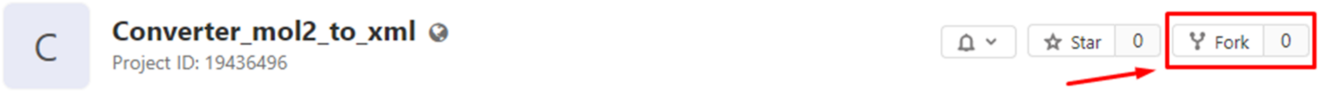

Figure S1. Clicking on the "Fork" button on the GitLab platform.

To run the code, install the development environment. The recommended environment is the "IntelliJ IDEA" (https://www.jetbrains.com/ru-ru/idea/).

Next, click on the converter_mol2_to_xml in a personal repository on the GitLab and copy the link (Figure S2).

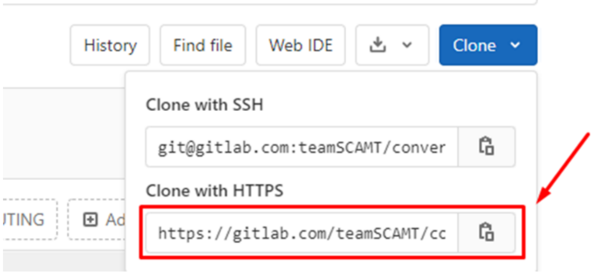

Figure S2. Cloning the HTTPS link.

After installation, open the program and create a new project from version control (Figure S3).

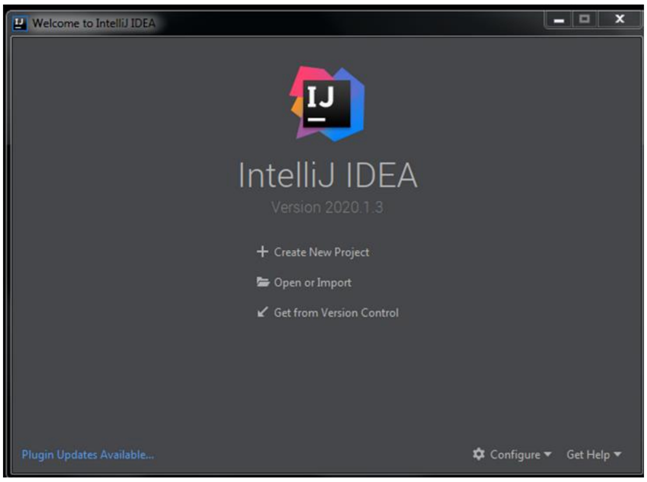

Figure S3. Creating a new project.

After that, paste the copied link in the URL line (Figure S4).

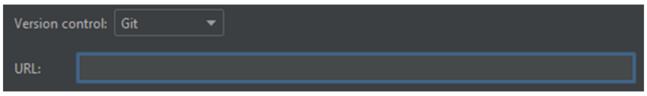

Figure S4. Pasting the copied link.

In order to run the code with the help of IntelliJ IDEA application, one needs to open all the three classes of the code: "Reader", "Beliviks" and "Writer"; this can be achieved by clicking on them.

As a result, the folder with the converter code will appear on the desktop. Next, add all Narupa Builder mol2 files, which require conversion to the folder with the converter code. Folder hierarchy is usually similar to the one given in Figure S5.

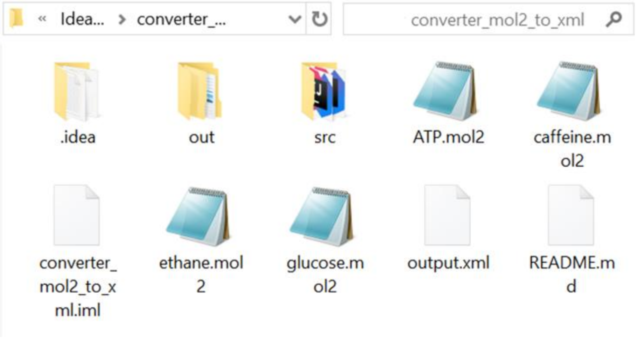

Figure S5. Folder hierarchy.

If everything is carried out correctly, the necessary mol2 file will appear in the IntelliJ IDEA between "Writer" and "README.md" classes (Figure S6).

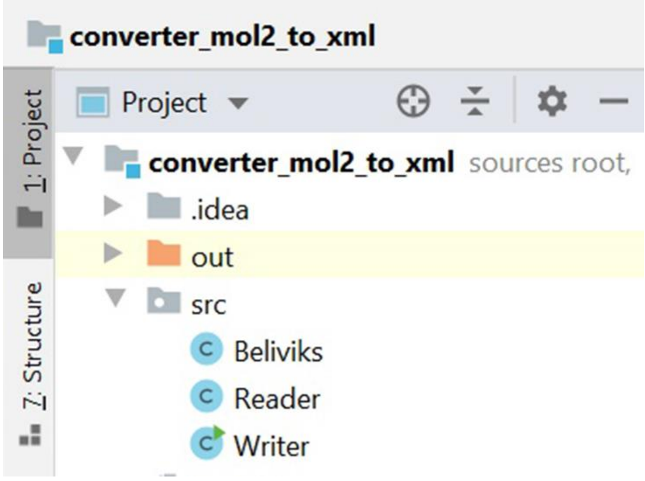

Figure S6. Adding the mol2 file obtained from Narupa Builder into the area where the finalized xml simulation files are opened.

Next, open class "Writer" and specify the name of the file, which requires conversion (an example is underlined in red in Figure S7).

```
public static void main(String[] args) throws ParserConfigurationException, 
    DocumentBuilderFactory dbFactory = DocumentBuilderFactory.newInstance();
    DocumentBuilder dBuilder = dbFactory.newDocumentBuilder();
    Document doc = dBuilder.newDocument();
    Reader read = new Reader(Paths.get( first: "ATP.mol2"));
```

Figure S7. Opening the "Writer" class and indicating the name of the desired mol2 file.

Finally, run the code, and the resulting xml code will appear in the output file. The code converted into the final xml file is presented below in Figure S8:

README.md Ethane.xml

```xml
<?xml version="1.0" encoding="utf-8"?>
<Simulation Name="c2h6">
  <SystemProperties>
    <SimulationBoundary SimulationBox="2, 2, 2" MinimumBoxVolume="2" />
    <Thermostat Type="BerendsenThermostat" EquilibriumTemperature="200" MaximumTemperature="10000" BerendsenCoupling="0.003" />
    <Integrator Type="VelocityVerlet" TimeStep="0.001" />
  </SystemProperties>
  <Topology>
    <Templates>
      <Residue Name="c2h6"> <!-- Created by Nadezhda Karandasheva March 16 2020-->
        <Atoms>
          <Atom Element="carbon" Position="-0.6165,0.0925,-0.6713" />
          <Atom Element="carbon" Position="-0.5555,0.0741,-0.5798" />
          <Atom Element="Hydrogen" Position="-0.7070,0.0516,-0.6599" />
          <Atom Element="Hydrogen" Position="-0.5711,0.0516,-0.7504" />
          <Atom Element="Hydrogen" Position="-0.6261,0.1910,-0.6857" />
          <Atom Element="Hydrogen" Position="-0.4737,0.0221,-0.6042" />
          <Atom Element="Hydrogen" Position="-0.6096,0.0221,-0.5137" />
          <Atom Element="Hydrogen" Position="-0.5287,0.1615,-0.5394" />
        </Atoms>
        <Bonds>
          <Bond A="0" B="1" />
          <Bond A="1" B="7" />
          <Bond A="1" B="6" />
          <Bond A="1" B="5" />
          <Bond A="0" B="4" />
          <Bond A="0" B="3" />
          <Bond A="0" B="2" />
         </Bonds>
        <ForceFields>
          <InteractiveGaussianForceField GradientScaleFactor="2000" />
          <MM3ForceField>
            <MM3AtomMappings>
              <MM3AtomMapping AtomPath="0" Type="1" />
              <MM3AtomMapping AtomPath="1" Type="1" />
              <MM3AtomMapping AtomPath="2" Type="5" />
              <MM3AtomMapping AtomPath="3" Type="5" />
              <MM3AtomMapping AtomPath="4" Type="5" />
              <MM3AtomMapping AtomPath="5" Type="5" />
              <MM3AtomMapping AtomPath="6" Type="5" />
              <MM3AtomMapping AtomPath="7" Type="5" />
            </MM3AtomMappings>
          </MM3ForceField>
          <LennardJonesForceField>
            <LennardJonesAtomMappings>
              <LennardJonesAtomMapping AtomPath="0" MM3Type="1" />
              <LennardJonesAtomMapping AtomPath="1" MM3Type="1" />
              <LennardJonesAtomMapping AtomPath="2" MM3Type="5" />
              <LennardJonesAtomMapping AtomPath="3" MM3Type="5" />
              <LennardJonesAtomMapping AtomPath="4" MM3Type="5" />
              <LennardJonesAtomMapping AtomPath="5" MM3Type="5" />
              <LennardJonesAtomMapping AtomPath="6" MM3Type="5" />
              <LennardJonesAtomMapping AtomPath="7" MM3Type="5" />
            </LennardJonesAtomMappings>
          </LennardJonesForceField>
        </ForceFields>
      </Residue>
    </Templates>

    <Spawners>
      <Spawner Name="c2h6" Template="c2h6" Count="1" />
    </Spawners>
  </Topology>
</Simulation>
```

Figure S8. The example of the code converted into xml format.

In the final xml file code, there are parts that the user needs to fill in manually. These are responsible for the assignment of bond type and are given in **bold,** as can be seen in an example below. In order to assign the bond type, one needs a glossary, which is provided in the mm3.xml file in the \server\Assets\Data\ folder.

<MM3AtomMapping AtomPath="0" **Type=**" "/>
<MM3AtomMapping AtomPath="1" **Type=**" "/>
...
<MM3AtomMapping AtomPath="7" **Type=**" "/>
...
<LennardJonesAtomMapping AtomPath="0" **MM3Type=**" "/>
<LennardJonesAtomMapping AtomPath="1" **MM3Type=**" "/>
...
<LennardJonesAtomMapping AtomPath="7" **MM3Type=**" "/>
